# Tradução, adaptação transcultural e validade de conteúdo da *Taxonomia das Propriedades de Medida* e da *Ferramenta COSMIN de Risco de Viés* para o Brasil

**DOI:** 10.1590/0102-311XPT025126

**Published:** 2026-10-12

**Authors:** Victor Hugo de Sousa Utida, Lunara Teles da Silva, Flavio Marques Lopes

**Affiliations:** 1 Pontifícia Universidade Católica de Goiás, Goiânia, Brasil.; 2 Universidade Federal de Goiás, Goiânia, Brasil.

**Keywords:** Estudos de Validação, Reprodutibilidade dos Testes, Viés, Validation Studies, Reproducibility of Results, Bias, Estudios de Validación, Reproducibilidad de los Resultados, Sesgo

## Abstract

A ausência de versões em português do Brasil da *Taxonomia das Propriedades de Medida* e da *Ferramenta COSMIN de Risco de Viés* limita a padronização da avaliação metodológica de estudos sobre confiabilidade e erro de medida no Brasil. O objetivo foi traduzir, adaptar transculturalmente e avaliar a validade de conteúdo dessas ferramentas para o contexto brasileiro. Estudo metodológico conduzido segundo diretrizes internacionais. O processo incluiu tradução dupla independente, síntese, retrotradução, apreciação técnica da equipe COSMIN e avaliação por especialistas por meio de Delphi modificado em duas rodadas. A validade de conteúdo foi examinada por abordagem quantitativa, com Índice de Validade de Conteúdo por Item (I-CVI) e o Coeficiente de Validade de Conteúdo (CVC), e qualitativa, com base em relevância, abrangência e compreensibilidade. Seis especialistas participaram da primeira rodada e cinco da segunda. Na primeira rodada, o I-CVI identificou problemas pontuais de clareza, e o CVC detectou um número maior de itens com necessidade de revisão. Os principais ajustes envolveram terminologia estatística, padronização textual e simplificação da redação. Após as revisões, todos os itens atingiram valores aceitáveis na segunda rodada. O CVC médio final foi de 0,915 para a *Taxonomia das Propriedades de Medida* e de 0,934 para a *Ferramenta COSMIN de Risco de Viés*. As versões em português do Brasil das ambas as ferramentas apresentaram evidências satisfatórias de validade de conteúdo, com adequação quanto à clareza, relevância, abrangência e compreensibilidade. Essas versões oferecem base metodológica padronizada para a avaliação de estudos sobre confiabilidade e erro de medida no contexto brasileiro.

## Abstract (en)

The absence of Brazilian Portuguese versions of the *Taxonomy of Measurement Properties* and the *COSMIN Risk of Bias Tool* limits the standardization of methodological evaluations in studies on reliability and measurement error in Brazil. This research aimed to translate, cross-culturally adapt, and evaluate the content validity of these tools for Brazil. This methodological study was conducted according to international guidelines. It included independent double translation, synthesis, back-translation, technical appreciation by the COSMIN team, and expert evaluation via two-round modified Delphi. Content validity was examined using a quantitative (item content validity index - I-CVI, and content validity coefficient - CVC) and qualitative approach (based on its relevance, comprehensiveness, and comprehensibility). In total, six experts participated in the first round and five in the second. In the first round, the I-CVI found problems of clarity, whereas the CVC detected a greater number of items in need of revision. Main adjustments involved statistical terminology, textual standardization, and writing simplification. After the revisions, all items reached acceptable values in the second round. The final mean CVC totaled 0.915 for the *Taxonomy of Measurement Properties* and 0.934 for the *COSMIN Risk of Bias Tool*. The Brazilian Portuguese versions of both tools showed satisfactory evidence of content validity, with adequate clarity, relevance, comprehensiveness, and comprehensibility. These versions provide a standardized methodological basis for evaluating studies on reliability and measurement error in the Brazilian context.

## Resumen

La falta de versiones en portugués brasileño de la *Taxonomía de las Propiedades de Medición* y de la *Herramienta COSMIN de Riesgo de Sesgo* limita la estandarización de la evaluación metodológica de los estudios sobre fiabilidad y error de medición en Brasil. Este estudio tuvo como objetivo traducir, adaptar de forma transcultural y evaluar la validez de contenido de estas herramientas para el contexto brasileño. El estudio metodológico se realizó siguiendo las directrices internacionales. El proceso incluyó doble traducción independiente, síntesis, retrotraducción, evaluación técnica por parte del equipo COSMIN y evaluación por expertos mediante el método Delphi modificado en dos rondas. La validez de contenido se examinó mediante enfoque cuantitativo, con el índice de validez de contenido por ítem (I-CVI) y el coeficiente de validez de contenido (CVC), y cualitativo, basado en la relevancia, la exhaustividad y la comprensibilidad. Seis expertos participaron en la primera ronda y cinco en la segunda. En la primera ronda, el I-CVI identificó problemas puntuales de claridad, mientras que el CVC detectó una mayor cantidad de elementos que necesitaban revisión. Los principales ajustes incluyeron la terminología estadística, la estandarización del texto y la simplificación de la redacción. Tras las revisiones, todos los elementos alcanzaron valores aceptables en la segunda ronda. El CVC promedio final fue de 0,915 para la *Taxonomía de las Propiedades de Medición* y de 0,934 para la *Herramienta COSMIN de Riesgo de Sesgo*. Las versiones en portugués brasileño de ambas herramientas presentaron evidencias satisfactorias de validez de contenido, demostrando adecuación con respecto a la claridad, la relevancia, la exhaustividad y la comprensibilidad. Estas versiones ofrecen una base metodológica estandarizada para evaluar estudios sobre fiabilidad y error de medición en el contexto brasileño.

## Introdução

A ausência de versões metodologicamente validadas, em português, da *Taxonomy, Terminology, and Definitions of Measurement Properties for Health-related Patient-reported Outcomes* (*Taxonomia das Propriedades de Medida*) ^1^ e da *COSMIN Risk of Bias Tool to Assess the Quality of Studies on Reliability and Measurement Error of Outcome Measurement Instruments* (*Ferramenta COSMIN Risco de Viés*) ^2^, tem limitado a padronização das revisões sistemáticas de propriedades de medida sobre confiabilidade e erro de medida no Brasil. Trata-se de uma deficiência estrutural que restringe o uso dessas ferramentas em sua integralidade e compromete a qualidade metodológica das evidências produzidas em língua portuguesa. Sem o acesso a instrumentos culturalmente adaptados, revisores acabam empregando critérios não padronizados, o que afeta a reprodutibilidade dos julgamentos, enfraquece a comparabilidade internacional das sínteses e obscurece decisões clínicas baseadas em evidência ^3^ ^,^ ^4^ ^,^ ^5^.

Instrumentos de medida em saúde precisam ser mais do que traduzidos. É necessário garantir equivalência conceitual, clareza terminológica e pertinência ao contexto clínico e cultural no qual serão aplicados ^5^ ^,^ ^6^ ^,^ ^7^. Ferramentas mal adaptadas ou com interpretações ambíguas comprometem a validade das inferências extraídas, e podem gerar recomendações clínicas distorcidas e políticas públicas mal sustentadas ^3^ ^,^ ^8^ ^,^ ^9^. Essa situação torna-se ainda mais sensível quando se trata de propriedades de medida como confiabilidade, erro de medida e consistência interna, cujos julgamentos dependem diretamente de critérios operacionais bem definidos ^10^ ^,^ ^11^ ^,^ ^12^.

A Prática Baseada em Evidências (PBE), como fundamento das tomadas de decisões em saúde, depende da qualidade dos instrumentos utilizados para a mensuração dos desfechos. Esses desfechos, sejam clínicos, funcionais, laboratoriais ou autorrelatados, devem ser medidos com rigor, especialmente quando utilizados em revisões sistemáticas que fundamentam diretrizes clínicas, recomendações regulatórias ou protocolos assistenciais ^4^ ^,^ ^9^ ^,^ ^11^ ^,^ ^13^ ^,^ ^14^. A ausência de instrumentos validados para contextos específicos, como o brasileiro, pode invalidar inferências críticas em avaliações de efetividade ou confiabilidade, mesmo quando os métodos estatísticos utilizados estejam corretos ^11^ ^,^ ^15^ ^,^ ^16^.

Nesse cenário, destaca-se a Iniciativa COSMIN (*Consensus-based Standards for the selection of health Measurement INstruments*), que consolidou uma taxonomia conceitual internacionalmente reconhecida e propôs ferramentas específicas para a avaliação da qualidade metodológica de estudos sobre propriedades de medida. Dentre essas, a *Ferramenta COSMIN Risco de Viés* (doravante denominada “*Ferramenta*”) ^2^ foi concebida para avaliar, com base em critérios objetivos, a qualidade de estudos que examinam confiabilidade e erro de medida de Instrumentos de Medida de Desfecho (OMI, acrônimo em inglês). Sua estrutura incorpora domínios como estabilidade da condição clínica, tempo entre medidas, aplicabilidade do delineamento e escolha dos métodos estatísticos, podendo ser utilizada tanto na prática clínica quanto em pesquisas científicas. Quando empregada em revisões sistemáticas, a Ferramenta permite julgamentos transparentes e padronizados, qualificando a seleção de instrumentos e reduzindo o risco de viés nas sínteses ^17^ ^,^ ^18^.

Apesar do crescente uso internacional das ferramentas COSMIN, sua aplicação em países de língua portuguesa ainda é limitada pela ausência de versões culturalmente adaptadas e semanticamente equivalentes. Essa lacuna não se restringe apenas à tradução linguística: envolve desafios conceituais, terminológicos e epistemológicos que precisam ser tratados com metodologia específica, sob pena de descaracterização dos critérios originais ^19^ ^,^ ^20^ ^,^ ^21^. Pesquisadores que empregaram versões não validadas de instrumentos em culturas distintas podem apresentar dificuldades operacionais e interpretações divergentes, que comprometem a consistência dos julgamentos e resultados ^7^ ^,^ ^21^ ^,^ ^22^ ^,^ ^23^. Isso evidencia a urgência de versões adaptadas transculturalmente, que respeitem a estrutura dos instrumentos originais e, ao mesmo tempo, sejam compreensíveis e aplicáveis pelos pesquisadores brasileiros.

A adaptação transcultural da *Taxonomia das Propriedades de Medida* (doravante denominada “*Taxonomia*”) ^1^ e da Ferramenta ^2^ exige um processo meticuloso, sendo pautado em diretrizes consolidadas como as de Beaton et al. ^24^, Terwee et al. ^5^, Cruchinho et al. ^7^, Eremenco et al. ^25^, que recomendam tradução direta e reversa, revisão por especialistas e avaliação de validade de conteúdo com base em métodos quantitativos e qualitativos. A validação não se limita à verificação semântica, pois exige que os itens do instrumento sejam julgados quanto à relevância, abrangência e compreensibilidade para o construto, a população-alvo e o contexto de uso ^5^ ^,^ ^6^ ^,^ ^26^. Essa abordagem é essencial para garantir que a versão final preserve a integridade metodológica dos instrumentos originais, mas sejam legítimas em seu novo contexto cultural.

Este estudo se dispôs a suprir essa lacuna, por meio da tradução, adaptação transcultural e avaliação da validade de conteúdo da Taxonomia e da Ferramenta. A versão resultante foi elaborada baseando-se em critérios metodológicos internacionalmente aceitos, avaliada por especialistas brasileiros experientes em clinimetria, psicometria e revisões sistemáticas de propriedades de medida. Visamos a suprir essa lacuna metodológica e ampliar a qualidade das revisões sistemáticas conduzidas no Brasil.

## Método

Trata-se de estudo metodológico de tradução, adaptação transcultural e avaliação da validade de conteúdo de instrumentos de medida, conduzido conforme referenciais internacionalmente reconhecidos para esse tipo de investigação, especialmente Beaton et al. ^24^, Terwee et al. ^5^ e Cruchinho et al. ^7^. O estudo foi aprovado pelo Comitê de Ética em Pesquisa da Universidade Federal de Goiás - UFG (CAAE 76485023.0.0000.5083; parecer nº 6.652.984), em conformidade com a *Resolução nº 466/2012* do Conselho Nacional de Saúde. Previamente à condução do estudo, a Iniciativa COSMIN concedeu autorização formal para o uso acadêmico das ferramentas.

O processo de adaptação transcultural seguiu cinco etapas, conforme Beaton et al. ^24^. Inicialmente, foram realizadas duas traduções independentes do inglês para o português do Brasil, por empresas de tradutores bilíngues com perfis distintos, sendo um com formação na área da saúde e outro sem formação técnica, com o objetivo de contemplar tanto a equivalência conceitual quanto a naturalidade linguística. As versões produzidas foram comparadas pela equipe de pesquisa, resultando em uma versão-síntese em português.

Em seguida, essa versão foi submetida a duas retrotraduções independentes, realizadas por tradutores bilíngues sem conhecimento do documento original. A síntese das retrotraduções, acompanhada da versão traduzida, foi encaminhada à equipe internacional da Iniciativa COSMIN para a apreciação técnica. Nessa etapa, o material foi examinado quanto à equivalência semântica, idiomática e conceitual em relação à versão original, bem como quanto à clareza, consistência terminológica e adequação do conteúdo ao construto avaliado. As observações recebidas subsidiaram o refinamento da versão traduzida antes da etapa de validade de conteúdo.

A validade de conteúdo foi avaliada por Delphi modificado, em duas rodadas, entre maio e agosto de 2024. Foram convidados 62 especialistas, dos quais seis aceitaram participar. A seleção dos participantes considerou: nível de pós-graduação e experiência consolidada em psicometria, clinimetria ou revisões sistemáticas, a experiência profissional, a produção científica na temática de interesse e a diversidade institucional e regional.

Cada especialista recebeu um dossiê eletrônico contendo os instrumentos originais, as versões traduzidas, as retrotraduzidas e as orientações para a avaliação. Os itens foram julgados em escala Likert de quatro pontos, variando de 1 (inadequado) a 4 (muito adequado). A apreciação contemplou equivalência semântica, idiomática e conceitual, além da clareza dos itens em português do Brasil. Em consonância com a COSMIN, esses julgamentos também subsidiaram a análise da relevância, da abrangência e da compreensibilidade do conteúdo ^5^ ^,^ ^6^. Para cada item, os especialistas registraram comentários qualitativos com sugestões e justificativas.

A análise da validade de conteúdo incluiu abordagens quantitativa e qualitativa, em conformidade com as recomendações da Iniciativa COSMIN para a avaliação da relevância, abrangência e compreensibilidade de instrumentos de mensuração de desfechos ^5^ ^,^ ^6^.

Na análise quantitativa, foram utilizados o Índice de Validade de Conteúdo por Item (I-CVI) e o Coeficiente de Validade de Conteúdo (CVC). O I-CVI foi calculado conforme Polit & Beck ^27^, pela razão entre o número de especialistas que atribuíram escores 3 ou 4 ao item e o número total de especialistas, segundo a fórmula *I-CVI = n*_*e*_ */N*, em que *n*_*e*_ corresponde ao número de especialistas que consideraram o item adequado e *N* ao número total de especialistas. Para painéis com seis ou mais especialistas, adotou-se como critério de aceitabilidade I-CVI ≥ 0,78 ^27^.

O CVC foi calculado segundo Hernández-Nieto ^28^. Para cada item, obteve-se inicialmente o CVCi com base na razão entre a média dos escores atribuídos pelos especialistas e o valor máximo da escala de resposta, conforme a expressão *CVCi = M*_*x*_ */V*_*max*_ . Em seguida, foi estimado o erro de polarização dos avaliadores pela fórmula *P*_*e*_ *= (1/J)* ^*J*^ , em que *J* representa o número de especialistas. O coeficiente corrigido de cada item foi calculado por *CVCc = CVCi - P*_*e*_ , e o coeficiente total do instrumento foi obtido pela média dos coeficientes corrigidos dos itens. Valores de CVC ≥ 0,80 foram considerados aceitáveis ^28^ ^,^ ^29^. O CVC foi adotado como medida contínua principal da validade de conteúdo por preservar a graduação das respostas dos especialistas, já o I-CVI foi utilizado como medida complementar, dada sua ampla utilização em estudos de validação de conteúdo ^28^ ^,^ ^29^ ^,^ ^30^ ^,^ ^31^ ^,^ ^32^.

Os comentários qualitativos dos especialistas foram avaliados segundo a análise de conteúdo de Bardin ^33^. O *corpus* foi constituído por todos os registros textuais produzidos nos formulários Delphi. A análise compreendeu pré-análise, exploração do material e tratamento dos resultados. Após a leitura flutuante, os comentários foram codificados e agrupados por unidades de sentido. As categorias analíticas foram definidas de forma dedutiva, com base nos domínios da validade de conteúdo adotados pela COSMIN ^5^, isto é, relevância, abrangência e compreensibilidade, sendo complementadas, quando pertinente, por aspectos de equivalência semântica, idiomática, conceitual e clareza linguística. A análise foi conduzida independentemente por dois revisores, com posterior consenso.

Após a incorporação dos ajustes decorrentes das análises quantitativa e qualitativa, foi elaborada a versão consolidada em português do Brasil. Essa versão foi novamente submetida à retrotradução e encaminhada para a apreciação final pela equipe internacional da Iniciativa COSMIN, a fim de verificar a manutenção da equivalência com a versão original, a adequação terminológica e a clareza da redação final. A presidente da Iniciativa COSMIN encaminhou a documentação a um membro brasileiro vinculado a COSMIN, a fim de subsidiar a análise técnica da versão traduzida. Baseando-se nesse processo, a equipe da Iniciativa COSMIN emitiu parecer contendo sugestões e considerações metodológicas, as quais orientaram o prosseguimento das etapas subsequentes de validação dos instrumentos.

As versões em português da Taxonomia e da Ferramenta estão publicamente disponíveis no sítio oficial da COSMIN (https://www.cosmin.nl/research-publications).

## Resultados

Foram convidados 62 especialistas para participar do estudo, dos quais seis responderam positivamente e compuseram o painel da primeira rodada do Delphi (taxa de resposta de 9,7%). Na segunda rodada, cinco especialistas permaneceram ativos. Todos possuíam experiência consolidada em clinimetria, psicometria ou revisões sistemáticas de propriedades de medida, com vinculação a universidades públicas situadas nas regiões Sudeste, Centro-oeste e Sul do Brasil. A média de idade dos participantes foi de 37,33 ± 7,20 anos, com predominância do sexo feminino (4; 66,7%). Em relação à titulação, 83,3% possuíam doutorado e a metade havia concluído estágio pós-doutoral. As características sociodemográficas e acadêmicas estão apresentadas na Tabela 1.

**Tabela 1:** Caracterizações sociodemográficas e acadêmicas dos especialistas no Delphi.

Variáveis	Média ± DP	Mediana
Idade (em anos)	37,33 ± 7,20	33,00
Tempo de formação (em anos)	15,00 ± 6,63	14,50
Variáveis	n (n = 6)	%
Formação profissional		
Fisioterapeuta	3	50,00
Farmacêutico(a)	2	33,33
Odontólogo(a)	1	16,67
Gênero		
Feminino	4	66,67
Masculino	2	33,33
Titulação		
Mestrado	1	16,67
Doutorado	2	33,33
Pós-doutorado	3	50,00

DP: desvio padrão.

Fonte: elaboração própria.

Na primeira rodada Delphi, a análise do I-CVI mostrou que a maior parte dos itens apresentou concordância satisfatória entre os especialistas. Entretanto, alguns resultados ficaram abaixo do ponto de corte recomendado para painéis com seis ou mais especialistas (I-CVI ≥ 0,78), especialmente na dimensão clareza. Na Taxonomia, domínio Confiabilidade: a versão estendida e o domínio Validade de construto obtiveram I-CVI de 0,667 para clareza. Na Ferramenta, o item 7 do domínio Confiabilidade: “Para pontuações contínuas: foi calculado um coeficiente de correlação intraclasse (ICC)?” e o item 7 do domínio Erro de medida: “Para pontuações contínuas: erro padrão da medida (SEM), a mínima mudança detectável (SDC), os limites de concordância (LoA) ou o coeficiente de variação (CV) foram calculados?” apresentaram I-CVI de 0,75 para clareza. Esses achados indicaram insuficiente concordância quanto à compreensibilidade de itens específicos e sinalizaram a necessidade de revisão textual.

A análise pelo CVC, por sua vez, foi mais sensível para detectar fragilidades na redação dos instrumentos e identificou um número maior de itens com desempenho insatisfatório. Na Taxonomia, a versão estendida do domínio Confiabilidade apresentou CVC abaixo de 0,80 em todas as dimensões avaliadas, com valores de 0,66 para equivalência semântica, 0,79 para equivalência idiomática, 0,75 para equivalência conceitual e 0,70 para clareza. A definição curta desse mesmo domínio apresentou CVC de 0,79 em todas as dimensões, e o domínio Validade de construto registrou CVC de 0,70 para clareza.

Na Ferramenta, também foram identificados itens com necessidade de revisão, incluindo o item 4 do domínio Confiabilidade: “O(s) profissional(is) administrou(ram) a mensuração sem conhecimento das pontuações ou valores de outra(s) medida(s) repetidas(s) nos mesmos pacientes?” e o item 4 do domínio Erro de medida: “O(s) profissional(is) administrou(ram) a mensuração sem conhecimento das pontuações ou valores de outra(s) medida(s) repetidas(s) nos mesmos pacientes?”, ambos com CVC de 0,75 para equivalência semântica, além do item 7 desses dois domínios: “Para pontuações contínuas: foi calculado um coeficiente de correlação intraclasse (ICC)?” e “Para pontuações contínuas: erro padrão da medida (SEM), a mínima mudança detectável (SDC), os limites de concordância (LoA) ou o coeficiente de variação (CV) foram calculados?”, com CVC de 0,75 para equivalência idiomática.

Em conjunto, os resultados mostraram que, embora o I-CVI tenha identificado problemas pontuais, o CVC apresentou maior capacidade discriminativa ao captar variações mais sutis na avaliação dos especialistas, exigindo a reavaliação de um número mais amplo de itens. Com base nesses achados, todos os itens com I-CVI < 0,78 ou CVC < 0,80 foram reformulados antes da segunda rodada Delphi. As modificações incluíram ajustes semânticos, gramaticais e conceituais, simplificação de estruturas excessivamente longas, padronização de siglas internacionalmente reconhecidas, como ICC, SEM, SDC, LoA e CV, e inclusão de explicações complementares na legenda da Ferramenta, com o objetivo de aprimorar a clareza, a precisão terminológica e a equivalência da versão em português do Brasil.

Na segunda rodada Delphi, os especialistas reavaliaram completamente as versões ajustadas e todos os itens atingiram valores de I-CVI e CVC iguais ou superiores a 0,80 em todas as dimensões, confirmando a equivalência semântica, idiomática, conceitual e de clareza das versões brasileiras dos instrumentos. O CVC médio da Taxonomia foi de 0,915, e a Ferramenta alcançou CVC médio de 0,934. Alguns domínios, como Teste de hipóteses e Responsividade, mantiveram índices máximos (CVC = 1,00) desde a primeira rodada. Os domínios Confiabilidade e Validade de Construto, e os itens 4 e 7 da Ferramenta, inicialmente problemáticos, atingiram pontuações aceitáveis após os ajustes realizados, conforme apresentado nas Tabelas 2 e 3.

**Tabela 2:** Coeficientes de Validade de Conteúdo (CVC) da versão brasileira da *Taxonomia das Propriedades de Medida*.

	CVC				
	Equivalência semântica	Equivalência idiomática	Equivalência conceitual	Clareza	Médio
Domínio: Confiabilidade	0,833	0,875	0,875	0,833	0,854
Confiabilidade: Versão estendida	0,950	1,000	1,000	0,900	0,962
Propriedade de medida: Consistência interna	0,833	0,833	0,833	0,833	0,833
Propriedade de medida: Confiabilidade	0,950	0,950	0,950	0,900	0,937
Propriedade de medida: Erro de medida	0,875	0,917	0,917	0,875	0,896
Domínio: Validade	0,875	0,833	0,875	0,875	0,865
Propriedade de medida: Validade de conteúdo	0,875	0,875	0,875	0,875	0,875
Aspectos de uma propriedade de medida: Validade de face	0,875	0,875	0,875	0,833	0,865
Propriedade de medida: Validade de construto	0,950	0,950	1,000	0,950	0,962
Aspectos de uma propriedade de medida: Validade estrutural	0,875	0,917	0,917	0,875	0,896
Aspectos de uma propriedade de medida: Teste de hipótese	1,000	1,000	1,000	1,000	1,000
Aspectos de uma propriedade de medida: Validade transcultural	0,875	0,917	0,917	0,875	0,896
Propriedade de medida: Validade de critério	0,875	0,917	0,917	0,917	0,906
Domínio: Responsividade	0,917	0,958	0,958	0,958	0,948
Propriedade de medida: Responsividade	1,000	1,000	1,000	1,000	1,000
Domínio: Interpretabilidade	0,958	0,958	0,917	0,917	0,937
CVC médio	0,907	0,923	0,926	0,901	0,915

Fonte: elaboração própria.

**Tabela 3:** Coeficientes de Validade de Conteúdo (CVC) da versão brasileira da *Ferramenta COSMIN de Risco de Viés* para estudos de confiabilidade e erro de medida.

	CVC				
	Equivalência semântica	Equivalência idiomática	Equivalência conceitual	Clareza	Médio
Confiabilidade					
Item 1	0,917	0,875	0,917	0,875	0,896
Item 2	0,917	0,958	0,958	0,917	0,937
Item 3	0,875	0,917	0,917	0,917	0,906
Item 4	1,000	1,000	1,000	1,000	1,000
Item 5	0,833	0,958	0,958	0,958	0,927
Item 6	0,958	0,958	0,958	0,917	0,948
Item 7	1,000	1,000	1,000	0,850	0,938
Item 8	0,875	0,958	0,958	0,958	0,937
Item 9	0,917	0,917	0,917	0,875	0,906
Erro de medida					
Item 1	0,833	0,833	0,875	0,875	0,854
Item 2	0,917	0,958	0,958	0,917	0,937
Item 3	0,875	0,917	0,917	0,917	0,906
Item 4	1,000	1,000	1,000	1,000	1,000
Item 5	0,800	0,917	0,917	0,917	0,885
Item 6	0,958	0,958	0,958	0,958	0,958
Item 7	1,000	1,000	1,000	0,850	0,938
Item 8	1,000	1,000	1,000	1,000	1,000
CVC médio	0,922	0,949	0,953	0,912	0,934

Fonte: elaboração própria.

A análise qualitativa das respostas abertas foi organizada de acordo com os três eixos centrais da Validade de conteúdo (relevância, abrangência, compreensibilidade) definidos por Terwee et al. ^5^. Os comentários dos especialistas foram incorporados literalmente sempre que possível, de modo a evidenciar com clareza as contribuições específicas que orientaram as revisões entre as rodadas Delphi.

No eixo da relevância, os especialistas não questionaram a pertinência geral dos itens em relação aos construtos avaliados, confirmando que todos os domínios são essenciais para avaliar propriedades de medida em saúde. No entanto, alguns comentários ressaltaram a necessidade de maior precisão terminológica em termos estatísticos, de modo a garantir aderência conceitual às definições originais. Um dos especialistas observou: “*No meu entender o termo ‘variação’ deveria ser substituído por ‘variância’*”, destacando que o ICC é fundamentado em variâncias e não em um uso genérico da palavra variação. Essa observação reforça o papel do rigor conceitual como componente da relevância, uma vez que a precisão terminológica assegura que a versão brasileira preserve integralmente a intenção estatística e metodológica dos itens originais. Outro painelista sugeriu a troca de “*capacidade*” por “*habilidade*” (“*Prefiro habilidade em vez de capacidade*”), considerando que o termo “capacidade” poderia gerar sobreposição com a Classificação Internacional de Funcionalidade, já “habilidade” reflete melhor a ideia de competência técnica. Esses comentários indicam que, embora os itens fossem reconhecidos como relevantes, ajustes finos eram indispensáveis para resguardar a integridade conceitual.

No eixo da abrangência não foram identificadas lacunas de conteúdo que comprometessem a completude da Taxonomia ou da Ferramenta. Os especialistas reconheceram que os domínios essenciais estavam contemplados e não recomendaram a inclusão de novos itens. Contudo, reforçaram a importância de uniformizar terminologias para garantir a cobertura metodológica de todas as métricas internacionalmente aceitas. Um exemplo foi a sugestão: “*Sugiro traduzir ‘study design’ por desenho/delineamento de estudo*”, evidenciando a necessidade de utilizar um termo consagrado na literatura nacional e internacional para manter a comparabilidade com pesquisas prévias. A recomendação de explicitar uma legenda com métricas clássicas como SEM, SDC, LoA e CV também surgiu nesse eixo, com a intenção de favorecer a completude e a aplicabilidade prática do instrumento sem perda de rigor metodológico.

Foi no eixo da compreensibilidade que foi concentrada a maior parte dos comentários qualitativos, refletindo preocupações com clareza, inteligibilidade e padronização linguística. Um especialista recomendou: “*Substituir medições por medida*”, destacando a importância de evitar redundância e manter a coerência terminológica. Outro apontou a ausência de elementos essenciais em alguns enunciados, sugerindo: “*Na versão em português faltou a palavra ‘repetida’*”, o que poderia comprometer a lógica do item e dificultar a sua interpretação. Também houve recomendação de ajuste no sujeito das frases: “*Substituir revisor por avaliador ou examinador*”, de modo a alinhar a terminologia à prática clínica e metodológica brasileira. Além disso, observou-se a necessidade de simplificação textual, com a anotação: “*Sugiro simplificar a frase*”, em referência a construções excessivamente longas e literais que, embora semanticamente corretas, poderiam comprometer a fluidez em português. Essas sugestões de simplificação não alteraram o conteúdo conceitual, mas aumentaram a inteligibilidade, reforçando que a clareza é um pré-requisito para a validade de conteúdo.

## Discussão

A principal contribuição deste estudo foi disponibilizar versões em português do Brasil da *Taxonomia das Propriedades de Medida* ^1^ e da *Ferramenta COSMIN de Risco de Viés* ^2^, com evidências de validade de conteúdo obtidas por processo de tradução e adaptação transcultural compatível com referenciais internacionais ^5^ ^,^ ^6^ ^,^ ^7^ ^,^ ^24^ ^,^ ^27^. A relevância desse achado decorre do fato de que, na perspectiva COSMIN, a validade de conteúdo é considerada a propriedade de medida mais fundamental, pois examina se os itens são relevantes, abrangentes e compreensíveis para o construto e para o contexto de uso ^5^ ^,^ ^6^.

Os achados deste estudo sugerem que a estrutura conceitual dos instrumentos originais foi preservada, mas que sua transferência para o português do Brasil exigiu refinamentos linguísticos pontuais, sobretudo nos itens relacionados à confiabilidade, ao erro de medida e à terminologia estatística. O dado mais importante aqui não é apenas que houve itens abaixo do ponto de corte na primeira rodada, mas onde essas fragilidades estavam concentradas: predominantemente na clareza e na formulação linguística, e não na pertinência global dos domínios. Essa distribuição é coerente com a literatura de adaptação transcultural, em que instrumentos metodológicos e de mensuração frequentemente demandam ajustes terminológicos finos para preservar a equivalência semântica e conceitual sem comprometer a inteligibilidade no idioma-alvo ^6^ ^,^ ^19^ ^,^ ^22^ ^,^ ^24^.

O fato de os problemas da primeira rodada terem se concentrado majoritariamente na clareza, e não na pertinência global dos domínios, reforça a interpretação de que não houve ruptura conceitual relevante, mas sim a necessidade de lapidação redacional para tornar explícito, em português do Brasil, o significado técnico originalmente pretendido. Essa leitura é consistente com a noção de que a adaptação transcultural bem-sucedida não se reduz à tradução literal, mas depende da manutenção do significado em contexto cultural e linguístico específico ^24^.

A comparação entre I-CVI e CVC merece destaque porque ela esclarece um ponto metodológico central do estudo. No presente conjunto de dados, os dois coeficientes convergiram ao apontar a necessidade de revisão; o I-CVI foi útil para identificar itens com discordância mais evidente, mas o CVC mostrou maior sensibilidade para detectar fragilidades graduais na redação dos itens. Isso é plausível porque o I-CVI opera com base em uma lógica dicotomizada, ao passo que o CVC preserva a graduação dos escores atribuídos pelos especialistas ^28^ ^,^ ^29^ ^,^ ^30^ ^,^ ^31^.

Essa diferença deve ser interpretada como uma característica deste conjunto de dados, e não como prova universal de superioridade de um índice sobre o outro. O I-CVI permanece amplamente utilizado para identificar a concordância quanto à adequação dos itens, mas sua lógica dicotomizada tende a ser menos sensível a variações graduais dos julgamentos ^27^. Já o CVC preserva a variação dos escores atribuídos pelos especialistas e, por isto, pode captar fragilidades mais sutis de redação e equivalência, particularmente em painéis pequenos ^28^ ^,^ ^29^ ^,^ ^30^. Assim, a principal força metodológica do estudo não foi escolher um coeficiente “melhor”, mas combinar duas abordagens complementares e demonstrar, empiricamente, que o CVC foi mais discriminativo nesta amostra específica.

Outro aspecto que precisa ser interpretado com sobriedade é o tamanho do painel de especialistas. Embora o número de participantes não permita classificar o estudo no nível metodológico mais alto sugerido por alguns referenciais, que consideram “muito bom” quando cada item é avaliado por sete ou mais participantes; “adequado” quando por cinco a seis; e “duvidoso” quando apenas três a quatro contribuem ^5^. As diretrizes da COSMIN destacam que a diversidade e a experiência temática dos avaliadores, somadas a um processo de julgamento transparente, podem ser mais relevantes do que o número absoluto, desde que haja suficiência para atingir a saturação ^6^.

Ainda assim, um painel reduzido restringe a estabilidade das estimativas e exige interpretação mais cautelosa dos coeficientes. Nesse sentido, a articulação entre análise quantitativa e análise qualitativa foi um ponto forte do estudo porque permitiu não apenas identificar itens problemáticos, mas compreender a natureza das dificuldades encontradas e orientar revisões textuais com fundamento técnico. Portanto, os resultados obtidos conferem suporte à validade de conteúdo da versão brasileira, mas não encerram o processo de avaliação das propriedades da ferramenta em seu novo contexto de uso ^5^ ^,^ ^6^ ^,^ ^10^ ^,^ ^32^.

A análise qualitativa ajudou a esclarecer a natureza das revisões necessárias. De modo geral, as sugestões dos especialistas foram concentradas em ajustes de redação, terminologia e organização das frases, e não em mudanças no conteúdo dos domínios ou itens. Isso reforça a interpretação de que a estrutura conceitual dos instrumentos foi preservada, mas alguns trechos ainda precisavam ser aprimorados para garantir maior clareza e uniformidade de interpretação no português do Brasil. Em instrumentos metodológicos, esse ponto é particularmente importante porque pequenas imprecisões de linguagem podem repercutir no julgamento da qualidade de estudos primários e, consequentemente, na consistência das revisões sistemáticas que utilizam essas ferramentas ^5^ ^,^ ^6^ ^,^ ^10^.

No eixo da relevância, os especialistas confirmaram a pertinência dos domínios e itens em relação ao construto avaliado. Os ajustes sugeridos tiveram caráter pontual e buscaram aumentar a precisão conceitual de termos técnicos, sem modificar o conteúdo original ^5^ ^,^ ^11^ ^,^ ^22^. No eixo da abrangência não foram identificadas lacunas que comprometessem a completude da Taxonomia ou da Ferramenta, o que indica que os elementos essenciais foram mantidos na versão brasileira. A preservação explícita das métricas clássicas de confiabilidade e erro de medida, como SEM, SDC, LoA, CV e ICC, reforça a integridade metodológica e garante comparabilidade internacional ^11^. Versões excessivamente adaptadas ao idioma local podem reduzir a precisão técnica dos instrumentos ^22^, por isto, optou-se por manter a estrutura original e complementar a versão brasileira com legendas explicativas, facilitando o uso por revisores nacionais sem descaracterizar o conteúdo. A inclusão da tradução integral do manual de uso prático ampliou a acessibilidade e atendeu à recomendação de diferentes autores quanto à necessidade de suporte técnico para o uso consistente das ferramentas ^10^ ^,^ ^17^.

As principais dificuldades concentraram-se, portanto, no eixo da compreensibilidade. Isso explica por que os itens com o pior desempenho na primeira rodada estavam relacionados sobretudo à clareza textual. Após a revisão da redação, simplificação de trechos longos e padronização de termos estatísticos, os itens reavaliados atingiram valores adequados na segunda rodada. Esse resultado sugere que os ajustes realizados melhoraram a inteligibilidade da versão brasileira sem comprometer a fidelidade ao instrumento original, o que está de acordo com as recomendações internacionais para a adaptação transcultural.

Entre os pontos fortes deste estudo, destacamos a adesão estrita às etapas de adaptação transcultural recomendadas, o parecer técnico externo da equipe COSMIN durante a retrotradução e a documentação transparente das revisões realizadas entre as rodadas, com incorporação literal das sugestões de especialistas. Como limitações, a amostra de especialistas não atingiu o nível “muito bom” e a avaliação de pré-teste e aplicabilidade prática será explorada em estudo derivado. Essa escolha de modular o processo de validação é compatível com práticas recomendadas por outros autores e recomendações internacionais, que reconhecem a necessidade de fases sucessivas para a validação de instrumentos complexos ^5^ ^,^ ^7^.

## Conclusão

Este estudo cumpriu com rigor o objetivo de traduzir, adaptar transculturalmente e reunir evidências de validade de conteúdo da *Taxonomia das Propriedades de Medida* e da *Ferramenta COSMIN de Risco de Viés* para o contexto brasileiro. As versões finais mostraram-se conceitualmente equivalentes aos instrumentos originais, com clareza, pertinência, abrangência e compreensibilidade adequadas após os ajustes realizados ao longo do processo de avaliação por especialistas.

As versões brasileiras podem contribuir para a maior padronização da avaliação metodológica de estudos sobre confiabilidade e erro de medida, com potencial para qualificar revisões sistemáticas e pesquisas sobre propriedades de medida em saúde. Adicionalmente, um estudo complementar de aplicabilidade prática e confiabilidade entre avaliadores foi conduzido com o objetivo de ampliar a avaliação da versão brasileira e fortalecer o seu uso em diferentes contextos de pesquisa. Assim, as versões brasileiras disponibilizadas neste estudo representam um passo metodológico relevante para qualificar a produção e a síntese de evidências sobre propriedades de medida no Brasil.

## Data Availability

Os dados de pesquisa estão disponíveis mediante solicitação ao autor de correspondência.
